# Cutibacterium avidum Osteomyelitis Associated With Pediatric Hip Reconstruction

**DOI:** 10.7759/cureus.42864

**Published:** 2023-08-02

**Authors:** Austin L Shiver, Styles Bertrand

**Affiliations:** 1 Orthopedics, Augusta University Medical College of Georgia, Augusta, USA

**Keywords:** dega osteotomy, neuromuscular hip dysplasia, cerebral palsy, varus derotational osteotomy, cutibacterium avidum

## Abstract

*Cutibacteria *are gram-positive, non-sporulating, anaerobic, or microaerophilic bacilli that are increasingly recognized in the setting of indolent post-operative infection. Clinically significant infection with *Cutibacterium avidum* in the pediatric population is rarely encountered. Herein, we report our experience with two pediatric cases of osteomyelitis and soft tissue abscess after femoral derotational osteotomy for congenital hip dysplasia.

## Introduction

Infection with *Cutibacterium *species is increasingly recognized as a source of late presenting and indolent infection. *Cutibacterium acnes* in particular has demonstrated a predilection for infection in arthroplasty of the shoulder. Cutibacteria are gram-positive, non-spore-forming, anaerobic, or microaerophilic bacilli that are members of normal skin flora [1]. *Cutibacterium* *avidum* is a recognized member of normal skin flora that resides in the sebaceous glands, and colonization increases with age similar to *Cutibacterium acnes*. It has been described as a source of septic arthritis [2], splenic abscess [3], sacroiliitis/osteomyelitis [4], and breast abscess [5], and is being increasingly recognized in adult periprosthetic hip infection [6]. We present our experience with two cases of infection with *Cutibacterium avidum* in two pediatric patients. There is little published literature on *Cutibacterium avidum* infection in the pediatric population, and to our knowledge, this is the first report of *Cutibacterium avidum* osteomyelitis after femoral de-rotational osteotomies of the hip.

## Case presentation

Case 1

**Figure 1:**
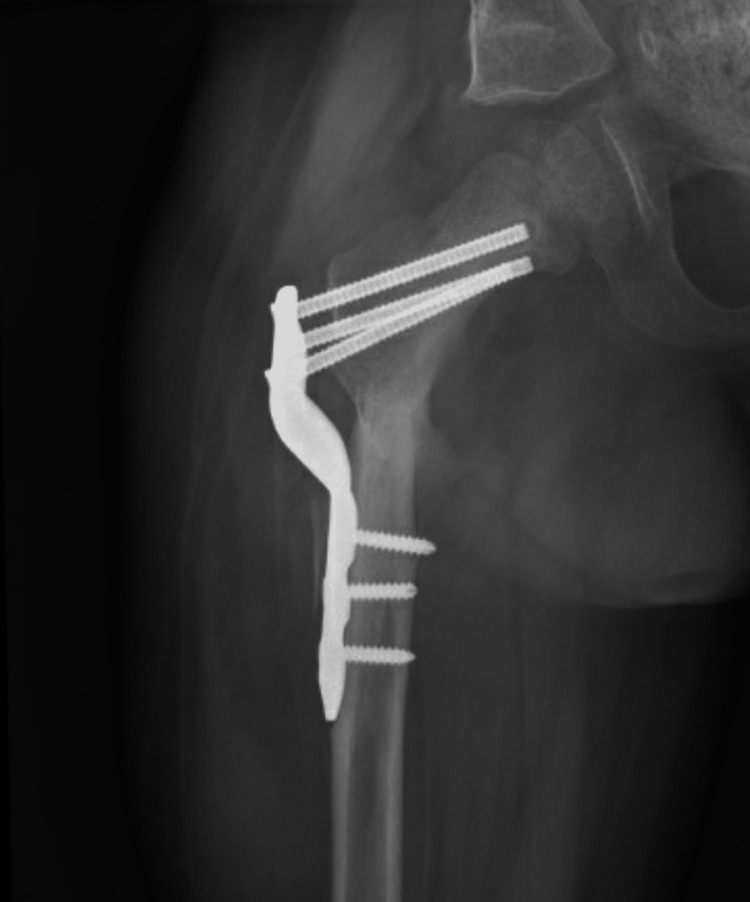
Anteroposterior view of the right hip

**Figure 2:**
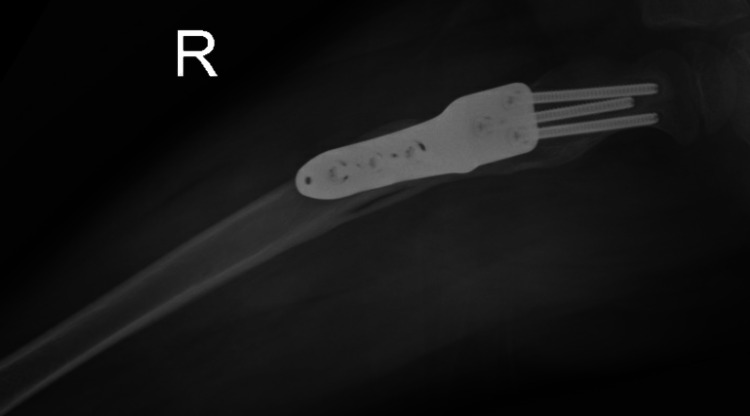
Frog lateral view of the right hip

A five-year-old African American male with a past medical history of 24-week prematurity, hydrocephalus, seizure disorder, and spastic quadriplegia presented to our clinic for the evaluation of multiple soft tissue contractures, foot deformity, and restricted hip range of motion. Upon evaluation, radiographs demonstrated neuromuscular hip dysplasia with a disruption of Shenton’s line in the right hemi-pelvis and coxa valga bilaterally. Decision was made for soft tissue release and hip containment. He underwent bilateral hamstring releases, bilateral Achilles tendon release, right adductor release, and left toe flexor release for contracture, and a right femoral varus derotational osteotomy with a Dega acetabular osteotomy. The immediate post-operative course was uncomplicated, and radiographs five weeks from index surgery demonstrated healing of the femoral osteotomy and no hardware complications. The patient began developing breakthrough seizures five months from the index surgery and subsequently required two admissions to the pediatric intensive care unit for seizure management. He was lost to follow-up and then represented 26 months after the index surgery to our hospital with a nodule and warmth underneath the healed incision. Examination revealed moderate thigh swelling, induration, erythema, and warmth and fluctuance to the distal aspect of the incision with tenderness to palpation. Radiographs at the time demonstrated no hardware failure, a healed proximal femoral osteotomy with surrounding soft tissue swelling, and hypodensity lateral to the proximal femoral hardware (Figures 1, 2).

Laboratory evaluation revealed a white blood cell count of 7,600/mm^3^ (reference range: 4,500-11,000/mm^3^), a differential including significant for 17% monocytes, a Westergren erythrocyte sedimentation rate of 20 mm/hour, and a C-reactive protein of 7.008 mg/dL (Table 1).

**Table 1:** Laboratory evaluation of case 1 WBC, white blood cell; ESR, erythrocyte sedimentation rate; CRP, C-reactive protein

Laboratory Evaluation		Reference
WBC count	7,600/mm^3^	4,500-1,1000/mm^3^
ESR	20 mm/hr	<20 mm/hour
CRP	7.008 mg/dL	<0.100 mg/dL
Gram stain	Negative	

He was admitted to the hospital and underwent irrigation and debridement of the surgical site with removal of surgical hardware. Operative findings included induration of the subcutaneous tissues and purulent fluid underneath the fibrous capsule overlying the proximal femoral plate; purulence was noted to express from the femur with removal of the three most proximal screws.

After deep cultures were obtained, empiric treatment with IV clindamycin 210 mg (10 mg/kg) every 8 hours was begun. This was changed to IV vancomycin 320 mg (15 mg/kg) every 8 hours on post-operative day 4 secondary to increasing C-reactive protein on post-operative day 3 and initial report of gram-positive bacilli. Initial gram stain demonstrated no neutrophils, no squamous cells, and no organisms. Three of the four obtained cultures demonstrated coryneforms (diphtheroids). Biopsy of the proximal femur was consistent with osteomyelitis. The cultures were referred to another laboratory, and the patient was discharged on IV vancomycin via peripherally inserted central catheter secondary to laboratory response. Further evaluation demonstrated *Cutibacterium avidum*, which was beta-lactamase negative with a penicillin MIC (minimum inhibitory concentration) of <0.5. He had clinical and laboratory response to vancomycin and received three weeks of IV vancomycin prior to finishing six weeks of antibiotic treatment with penicillin 100 mg/kg/day.

Case 2

Our second case is of an eight-year-old Caucasian female with a past medical history of spastic quadriplegia, neuromuscular hip dysplasia, and neuromuscular scoliosis, who had been previously followed by our clinic; the patient presented with complaints of increasing adduction deformity of the left hip and limb length inequality. Examination revealed palpable subluxation with reduction when the hip was abducted. Radiographs at that time demonstrated a break in Shenton’s line on the left, coxa valga, and greater than 50% left femoral head subluxation. She was then taken to the operating room for varus derotational osteotomy of the left femur, Dega acetabular osteotomy, and bilateral adductor and psoas tendon releases. She did well in the peri-operative period and subsequently underwent a posterior spinal fusion for worsening neuromuscular scoliosis four months after the index left hip procedure. There were no peri-operative complications after posterior spinal fusion. Eight months from the index hip procedure, she presented to her primary care physician with pain to palpation over the former femoral osteotomy site and the presence of a nodular subcutaneous mass. She had no fever. She received a two-week course of oral clindamycin, and after her symptoms failed to resolve, she was admitted to an outside facility. She underwent superficial abscess debridement, received empiric treatment with intravenous vancomycin, and was transitioned to oral clindamycin after cultures were reported as negative. No culture results were available for review from the outside facility. She was then seen at our hospital four weeks from initial incision and drainage with continued drainage and was admitted for surgical exploration. Laboratory evaluation at time of admission revealed a white blood cell count of 8,500/mm^3^ with 70% segmented neutrophils, a Westergren erythrocyte sedimentation rate of 20 mm/hour, and a C-reactive protein of 1.235 mg/dL (Table 2).

**Table 2:** Laboratory evaluation of case 2 WBC, white blood cell; ESR, erythrocyte sedimentation rate; CRP, C-reactive protein

Laboratory Evaluation		Reference
WBC	8,500/mm^3^	4,500-1,1000/mm^3^
ESR	20 mm/hour	<20 mm/hour
CRP	1.235 mg/dL	<0.100 mg/dL
Gram stain	Negative	

**Figure 3:**
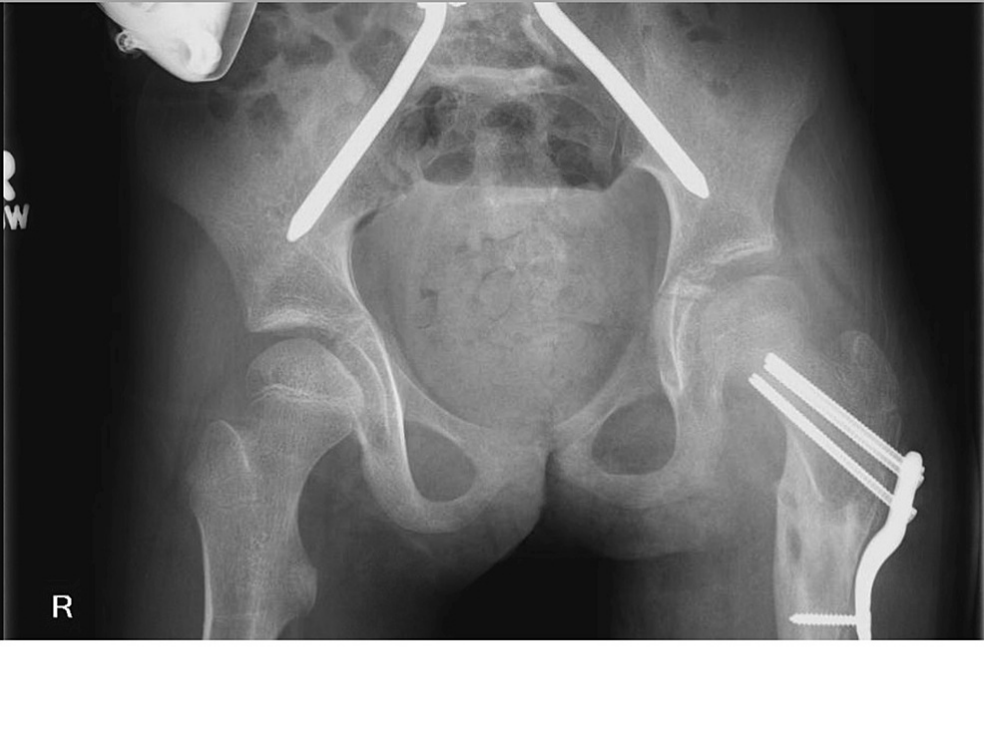
Anteroposterior view of the pelvis demonstrating left proximal femur sequestrum

Radiographs demonstrated a healed osteotomy with circumscribed lytic lesions with sclerosis along the medial femoral cortex at the level of the lesser trochanter concerning for sequestrum (Figure 3).

At the time of surgery, a sequestrum was encountered, removed, and sent for culture along with multiple tissue/fluid cultures. Three drains were place, and vancomycin 300 mg (20 mg/kg) every 6 hours was begun. She underwent repeat irrigation and debridement four days from the initial debridement procedure, and her wounds were closed. Initial culture results were reported as coryneforms (diphtheroids). Further testing revealed *Cutibacterium avidum* that was beta-lactamase negative, and vancomycin E-test of 0.5 demonstrating sensitivity. She was discharged on IV vancomycin 500 mg three times a day via a peripherally inserted central catheter. She received three weeks of intravenous therapy, which was then changed to amoxicillin 500 mg orally three times a day for a total therapy of seven weeks.

## Discussion

*Cutibacterium* species are known members of normal skin flora. In general, colonization with this species increases with increasing age [7]. The presence of *Cutibacterium* species is increasingly recognized as a source of infection in the orthopedic literature. The pathogenicity of *Cutibacterium* species has not been fully elucidated. It is known that *Cutibacterium acnes *is capable of producing extracellular proteinases, hyaluronidase, lipase, and biofilm [8]. Ascertaining *Cutibacterium avidum* as the causative agent can be difficult. Often, when found in blood or body fluid cultures, *Cutibacterium avidum* is often considered a contaminant [1]. In our case series, initial cultures were read as "diptheroids"; however, further study speciated the causative germ. Also, when considering for the question of contaminant, we recommend obtaining multiple cultures at the time of debridement to establish concurrence. *Cutibacterium avidum* is not known to cause primary infection; it results as a complication of invasive procedure or implanted hardware. As *Cutibacterium avidum* increases with age, it is rarely a cause of postoperative infection in childhood. In fact, we are unaware of a report in the literature involving pediatric patients. As our patients demonstrate, presentation is usually subacute with incubation lasting from days to months from primary procedure. *Cutibacterium avidum* is susceptible to common antibiotics save for metronidazole; review of the literature demonstrates typical treatment with beta-lactam agents as in our patients [8].

## Conclusions

*Cutibacterium* species should be considered as the source of infection and not as a contaminant when preceded by instrumentation. This family of bacteria is increasing recognized in the post-operative adult population. Our recent experience serves to demonstrate a pediatric incidence as well. In our cases, initial cultures revealed "diptheroids," which are considered contamination. In the appropriate setting, the treating surgeon should consider requesting further study and consideration for *Cutibacterium* species. In our limited series, both cases were treated with operative debridement with initial vancomycin followed by a penicillin agent.
